# Safety and toxicity of combined oclacitinib and carboplatin or doxorubicin in dogs with solid tumors: a pilot study

**DOI:** 10.1186/s12917-019-2032-4

**Published:** 2019-08-13

**Authors:** Laura E. Barrett, Heather L. Gardner, Lisa G. Barber, Abbey Sadowski, Cheryl A. London

**Affiliations:** 1 Cummings School, Tufts University, Foster Hospital for Small Animals, 200 Westboro Rd, N., Grafton, MA 01536 USA; 2 Sackler School of Graduate Biomedical Sciences, Tufts University, Boston, MA USA

**Keywords:** Oclacitinib, Carboplatin, Doxorubicin, Chemotherapy

## Abstract

**Background:**

Oclacitinib is an orally bioavailable Janus Kinase (JAK) inhibitor approved for the treatment of canine atopic dermatitis. Aberrant JAK/ Signal Transducer and Activator of Transcription (STAT) signaling within hematologic and solid tumors has been implicated as a driver of tumor growth through effects on the local microenvironment, enhancing angiogenesis, immune suppression, among others. A combination of JAK/STAT inhibition with cytotoxic chemotherapy may therefore result in synergistic anti-cancer activity, however there is concern for enhanced toxicities. The purpose of this study was to evaluate the safety profile of oclacitinib given in combination with either carboplatin or doxorubicin in tumor-bearing dogs.

**Result:**

Oclacitinib was administered at the label dose of 0.4–0.6 mg/kg PO q12h in combination with either carboplatin at 250-300 mg/m^2^ or doxorubicin at 30 mg/m^2^ IV q21d. Nine dogs were enrolled in this pilot study (*n* = 4 carboplatin; *n* = 5 doxorubicin). No unexpected toxicities occurred, and the incidence of adverse events with combination therapy was not increased beyond that expected in dogs treated with single agent chemotherapy. Serious adverse events included one Grade 4 thrombocytopenia and one Grade 4 neutropenia. No objective responses were noted.

**Conclusions:**

Oclacitinib is well tolerated when given in combination with carboplatin or doxorubicin. Future work is needed to explore whether efficacy is enhanced in this setting.

## Background

The Janus Kinase (JAK) and Signal Transducer and Activator of Transcription (STAT) protein families are important mediators of signal transduction for both cytokine and growth factor receptors involved in hematopoiesis and inflammation. Upon ligand binding to cytokine receptors and subsequent transphosphorylation, JAKs activate intracellular signaling cascades through phosphorylation of STATs, which then act as transcription factors for a variety of downstream targets. Specificity is determined by combinations of particular JAK (JAK1, JAK2, JAK3, TYK2) and STAT family members (STAT1, STAT2, STAT3, STAT4, STAT5a, STAT5b, STAT6), in addition to the cytokine or growth factor that initiated pathway activation [1]. Along with the role of JAK/STAT in driving normal cellular processes, STAT3, and to a lesser extent STAT5, are well defined contributors to tumor cell growth, while also promoting inflammation within the tumor microenvironment that supports disease progression [2–5].

Given its crucial role in promotion of angiogenesis, invasion and metastasis, immune evasion, and cell proliferation and survival, the JAK/STAT3 pathway is an attractive target for anti-neoplastic therapeutics. Several different STAT3 inhibitors have been evaluated in vitro and mouse models in vivo with variable success. Despite this progress, significant barriers to clinical translation remain including development of a STAT3 specific inhibitor with limited off-target effects, a good pharmacokinetic profile and low clinical toxicity [2, 6, 7]. Given that much of STAT activation is driven through JAKs, it is possible to manipulate the status of STAT phosphorylation through targeted inhibition of specific JAK family members. One JAK3 inhibitor, tofacitinib, (Xeljanz®, Pfizer), has received approval from the Food and Drug Administration (FDA) for the treatment of rheumatoid arthritis, psoriatic arthritis, and ulcerative colitis, and other JAK inhibitors are being investigated in a variety of immune-mediated inflammatory diseases in people [8, 9]. Ruxolitinib (Jakafi®, Incyte Corp.), a JAK1/JAK2 inhibitor, has been important in management of myeloproliferative neoplasms, such as myelofibrosis and polycythemia vera, in which gain-of-function mutations in JAK2 are found in 50–95% of patients [7]. And while constitutive STAT3 phosphorylation is common in many cancers, JAK inhibitors have had limited clinical efficacy as single agents for the treatment of advanced stage solid tumors in people thus far [7, 10, 11]. It is therefore likely that biologic activity of JAK/STAT inhibition in the setting of solid tumors will require combination with other therapeutic modalities, such as chemotherapy.

With respect to canine cancers, activation of JAK/STAT pathways have been documented in diffuse large B cell lymphoma, osteosarcoma, metastatic mammary carcinoma, and hemangiosarcoma [12–15] suggesting that as with some human cancers, blockade of this pathway may have biologic activity in dogs. Oclacitinib, a JAK inhibitor with significant structural similarities to tofacitinib, is FDA approved for the control of pruritus associated with allergic dermatitis and the control of atopic dermatitis in dogs over 12 months of age. Oclacitinib has preference for JAK1 inhibition over JAK2 at the recommended doses, causing downregulation of JAK1/JAK3 dependent cytokines (IL-2, IL-4, IL-6, IL-13, and IL-31), with minimal effects on JAK2-dependent cytokines (erythropoietin (EPO) and granulocyte/monocyte-colony stimulating factor (GM-CSF)) [16]. Oclacitinib has a favorable safety profile, though mild decreases in white blood cell and platelet counts have been documented [17–20]. Similarly, ruxolitinib is associated with dose-dependent thrombocytopenia and anemia in people with myelofibrosis [21, 22]. These are expected adverse events, as the JAK/STAT pathway is widely utilized by cytokines important in hematopoiesis, including GM-CSF, EPO and thrombopoietin (TPO) [23]. Given the emerging role of STAT3 in canine cancers such as osteosarcoma, there is interest in combining JAK/STAT inhibition (oclacitinib) with conventional chemotherapy. However, there was concern that inhibition of cytokine signaling critical for hematopoiesis would impair hematologic recovery from cytotoxic chemotherapy, particularly those known to cause myelosuppression, such as carboplatin and doxorubicin. Therefore, the purpose of this pilot study was to define the safety and tolerability of oclacitinib in conjunction with two standard chemotherapeutic drugs, carboplatin and doxorubicin, in dogs with solid tumors as a prelude to future clinical trials evaluating the therapeutic efficacy of this combination in defined tumor histologies.

## Methods

### Eligibility

This clinical trial was approved by the Institutional Animal Care and Use Committee at Tufts University and the Clinical Studies Review Committee at the Cummings School of Veterinary Medicine. Written informed consent was obtained from owners prior to clinical trial enrollment. Client owned dogs with cytologically and/or histologically confirmed carcinoma, sarcoma, or melanoma with measurable local or metastatic disease were eligible for enrollment. Prior chemotherapy, radiation therapy, or other investigational drug was allowable with a one-week washout period before enrollment. Inclusion criteria included age of at least one year, modified ECOG performance score of 0–1, absolute neutrophil count > 1000, platelet count > 80,000, total bilirubin < 1.5x the upper limit of normal, alanine transaminase <3x the upper limit of normal, serum creatinine < 1.5x the upper limit of normal, and no other serious systemic disorder considered incompatible with the study. Initial screening included physical examination, complete blood count, serum biochemistry profile, urinalysis and thoracic radiographs and/or abdominal ultrasound for evaluation of metastatic disease.

### Drug product and concomitant medications

Oclacitinib was provided by Zoetis (Parsippany, New Jersey) in 3.6 mg, 5.4 mg, and 16 mg tablets. The target dose of oclacitinib was 0.4–0.6 mg/kg orally every 12 h throughout the 42 day duration of the study. Concomitant medications acceptable for use included: metoclopramide, ondansetron, maropitant, famotidine, ranitidine, omeprazole, metronidazole, bismuth subsalicylate, loperamide, butorphanol, tramadol, fentanyl, diphenhydramine, and S-adenosylmethionine/silybin.

### Study design

The purpose of this study was to determine the safety and tolerability of oclacitinib in combination with chemotherapy. This was a pilot study with the intent to assess adverse events associated with drug combination, so no power calculation was made. Patients were prospectively enrolled into this trial and received either carboplatin (Group 1) or doxorubicin (Group 2), based upon clinician’s preference, tumor histology and prior chemotherapy treatment (carboplatin versus doxorubicin). Because several dogs has received prior chemotherapy, it was not possible to randomize dogs to the study groups. Dogs in Group 1 received carboplatin at a dose of 300 mg/m^2^ IV (or 250 mg/m^2^ for patients < 10 kg) once every 3 weeks for 2 cycles. Dogs in Group 2 received doxorubicin at a dose of 30 mg/m^2^ IV (or 1 mg/kg for patients < 10 kg) once every 3 weeks for 2 cycles. Oclacitinib was administered twice daily throughout the duration of the study in both groups.

Dogs were evaluated once weekly for the duration of this 42-day study. All dogs were evaluated on Days 0, 21, and 42 with physical examination, complete blood count, serum biochemistry profile, and either thoracic radiographs or abdominal ultrasound. Complete blood counts and physical examinations were also performed on Days 7, 14, 28, and 35 by clinicians at Tufts Cummings School of Veterinary Medicine or with the patient’s primary care veterinarian. Urinalyses were performed on Days 0 and 42. Owners completed a quality of life assessment survey weekly.

### Toxicity assessment

All adverse events were graded according to the published VCOG-CTCAE criteria, based upon a weekly quality of life assessment survey completed by clients, physical examination, complete blood count, chemistry profile, and urinalyses [24]. A chemotherapy dose reduction or delay was instituted in dogs experiencing Grade 3 or greater adverse events. The dose limiting toxicity was defined as a Grade 4 neutropenia or thrombocytopenia, Grade ≥ 3 non-hematologic toxicity with exception of ≥ Grade 3 diarrhea that did not resolve to ≤ Grade 2 within 48 h, and any Grade ≥ 3 ALT/AST elevation that did not resolve to ≤ Grade 2 within 7 days.

### Response assessment

Tumor response was assessed using the VCOG Response Evaluation Criteria for Solid Tumors in Dogs (v1.0) [25] and was evaluated at Day 21 and Day 42. A complete response (CR) was defined as complete regression of all measurable disease; partial response (PR) was defined as a > 30% reduction in the sum of the target lesion(s) longest diameter; stable disease (SD) was defined as a < 30% reduction or < 20% increase in the sum of the target lesion(s) longest diameter; and progressive disease (PD) was defined as a > 20% increase in the sum of the target lesion(s) longest diameter or the appearance of new lesions.

## Results

### Patient demographics

Nine dogs (*n* = 4 castrated male; *n* = 5 spayed female) were enrolled in the pilot study from May 2017 – December 2017. The median age was 10.8 years (range 8.0–14.8 years) and mean weight was 29.8 kg (range 8.6–69.2 kg). There were two greyhounds, two Huskies, two mixed breed dogs, one Leonberger, one Irish Wolfhound, and one Viszla. Four dogs were enrolled in Group 1 (carboplatin), consisting of patients with one each: recurrent anal sac adenocarcinoma, oral malignant melanoma with pulmonary metastasis, distal radial osteosarcoma without metastasis, and oral malignant melanoma. Group 2 (doxorubicin) consisted of four dogs with pulmonary metastasis from appendicular osteosarcoma and one dog with recurrent abdominal myxosarcoma. One patient in group 1 had a pre-existing grade 2 elevation in ALP at the start of the study. No other patients had abnormalities noted on pre-treatment complete blood count, serum chemistry profile or urinalysis.

### Treatment course

The starting dose of oclacitinib ranged from 0.25–0.53 mg/kg PO q12h, with a median dose of 0.43 mg/kg. One patient in Group 1 deviated from study protocol and received 0.25 mg/kg PO q12h for the duration of the patient’s time on study due to a mathematical error in dose calculation. In Group 1, three patients received carboplatin at a starting dose of 300 mg/m^2^ and one patient received carboplatin at a starting dose of 250 mg/^2^ due to a body weight of < 10 kg. This patient also required a carboplatin dose delay due to a neutrophil nadir at 21 days and was maintained on an every 4-week protocol with no reduction in dose. In Group 2, all patients were started at 30 mg/m^2^ doxorubicin IV every 3 weeks. Two patients required a dose reduction of doxorubicin due to Grade 3 and 4 neutropenia and no patients required a doxorubicin dose delay.

### Adverse events

The most common adverse events (AEs) were hematologic in origin (Table 1). Treatment was overall well tolerated, with low grade (Grade 1 and 2) liver transaminase elevations and hematologic AEs. One additional patient with a pre-existing Grade 2 elevation in ALP experienced no worsening in this parameter throughout the study. Serious adverse events included a Grade 4 neutropenia at 7 days post-doxorubicin and a Grade 4 thrombocytopenia at 14 days post-carboplatin. Gastrointestinal adverse events were rare. One Grade 3 anorexia was noted in conjunction with a Grade 4 neutropenia at 7 days post-doxorubicin chemotherapy in one patient.

**Table 1:** Treatment Emergent Adverse Events

Adverse event	Group 1				Group 2			
	Grade 1	Grade 2	Grade 3	Grade 4	Grade 1	Grade 2	Grade 3	Grade 4
Neutropenia	2	1			2		1	1
Thrombocytopenia	1			1	2		1	
Anemia	1				1			
Anorexia							1	
Diarrhea					1			
Increased ALP					2			
Increased ALT					1			
Increased AST					1			
Hypocalcemia					1			
Hypomagnesemia					1			

Two dogs were euthanized during the study period. One dog with metastatic oral melanoma developed pleural effusion on Day 3 of the carboplatin arm of the study and was euthanized without necropsy; this was suspected to be due to progression of preexisting pulmonary metastatic disease. Another dog was withdrawn by the owner and euthanized by the primary care veterinarian without evaluation on Day 21.

### Response to therapy

Response to therapy was a secondary objective of the study and was evaluated on Days 21 and 42. Table 2 details treatment and outcome for all dogs enrolled in the clinical study. Four patients successfully completed the study. Three patients were withdrawn due to progressive disease at day 21: two with progressive pulmonary metastasis from osteosarcoma and one with progressive local disease and suspected new development of pulmonary metastasis from an oral malignant melanoma. Two patients were euthanized during the study period: one at Day 3 due to development of pleural effusion attributed to progression of pulmonary metastasis from oral malignant melanoma; another was withdrawn at the owners’ request at Day 21, did not return for evaluation, and was euthanized by the primary care veterinarian.

**Table 2:** Cohort Demographics and Outcome

Dog	Signalment	Tumor	Prior Treatment(s)	Status at Enrollment	Study Treatment(s)	Outcome
1	13.6 yo MC Dachshund mixed breed	ASACA	Surgery, mitoxantrone × 8	Local recurrence, no metastasis	Carboplatin 250 mg/m^2^, 250 mg/m^2^	SD at Day 21, PD at Day 42
2	14.7 yo MC Husky	OMM	Surgery, melanoma vaccine, Palladia	Pulmonary metastasis	Carboplatin 300 mg/m^2^	Euthanized; decompensated on D3
3	8.0 yo FS Leonberger	OSA	None	Primary tumor	Carboplatin 300 mg/m^2^	Withdrawn at Day 21 without evaluation
4	11 yo MC mixed breed	OMM	None	Local tumor, pulmonary metastasis	Carboplatin 300 mg/m^2^	PD at Day 21
5	4.4yo MC Irish Wolfhound	OSA	Amputation, carboplatin × 2	Pulmonary metastasis	Doxorubicin 30 mg/m^2^	PD at Day 21
6	12.5yo FS Viszla	Abdominal Myxosarcoma	Surgery	Local recurrence	Doxorubicin 30 mg/m^2^, 25 mg/m^2^	SD at Day 21 SD at Day 42
7	10.8yo FS Greyhound	OSA	Amputation, carboplatin × 2	Pulmonary metastasis	Doxorubicin 30 mg/m^2^, 30 mg/m^2^	SD at Day 21 PD at Day 42
8	7.2 yo MC Greyhound	OSA	Amputation, carboplatin × 3	Pulmonary metastasis	Doxorubicin 30 mg/m^2^	PD at Day 21
9	10.6 yo FS Husky	OSA	Amputation	Pulmonary metastasis	Doxorubicin 30 mg/m^2^, 22.7 mg/m^2^	SD at Day 21 PD at Day 42

At Day 21, there were 7 dogs available for response evaluation. No complete or partial responses were noted at Day 21. Four dogs (57%) experienced SD, while 3 dogs demonstrated PD. At Day 42, there were four patients available for response evaluation, with 1 dog experiencing SD, and 3 dogs demonstrating PD. Of note, 6 of the 9 dogs treated had sarcoma/metastatic sarcoma which is notoriously resistant to chemotherapy, so a lack of response is not necessarily surprising.

## Discussion

The JAK/STAT pathway plays a critical role in oncogenesis, with constitutive activation leading to increased tumor cell proliferation, survival, invasion, and immunosuppression [7]*.* Drivers of JAK/STAT pathway activation include mutations in JAK family members, gain-of-function mutations in cytokine receptors, excessive autocrine or paracrine cytokine release within the tumor microenvironment and loss of negative regulation [26]. With respect to STAT3, constitutive is observed in > 70% of human cancers and has been found to be a negative prognostic indicator for several of these [27]. Moreover, in the tumor microenvironment, IL-6/JAK/STAT3 signaling acts to drive the proliferation, survival, invasiveness, and metastasis of tumor cells, while strongly suppressing the antitumor immune response [27]. As such, this pathway is an attractive target for the development of new therapies. Oclacitinib, an FDA-approved JAK inhibitor for the treatment of atopic dermatitis in dogs, blocks IL-6 signaling and subsequent STAT3 phosphorylation at recommended dosing [16], thus represents a viable approach to targeting STAT3 in veterinary cancer patients.

Hematologic toxicities are well-documented side effects of JAK inhibitors in people due to the crucial role of JAK/STAT signaling in hematopoiesis [23]. Anemia and thrombocytopenia are the most commonly reported hematologic adverse events in studies of the JAK2 inhibitor ruxolitinib in patients with myeloproliferative disorders, but rarely lead to treatment discontinuation and counts often stabilize during treatment [28]. Two long term extension studies of tofacitinib, (a JAK1/JAK3 inhibitor with a nearly identical target profile to that of oclacitinib), in patients with rheumatoid arthritis found that fewer than 5% experienced neutropenia of any degree, and most were mild in severity [29]. As monotherapy, oclacitinib is generally well tolerated, with gastrointestinal upset being the most commonly reported AE. When administered at the recommended label dosage, oclacitinib is predicted to have a minor impact on JAK2-dependent cytokines such as EPO and GM-CSF. However, while rarely leading to drug discontinuation, transient hematologic AEs (neutropenia and thrombocytopenia) have been reported in multiple studies [17–20]. Though hematologic toxicities are typically mild and self-limiting when JAK inhibitors are used as monotherapy, the potential for overlapping toxicity is a concern when these agents are combined with standard MTD chemotherapy.

The pilot study reported in this manuscript represents the first formal assessment of the safety and toxicity of oclacitinib given in combination with chemotherapy (doxorubicin or carboplatin) in tumor-bearing canine patients. Although two grade 4 hematologic toxicities were noted (grade 4 neutropenia with doxorubicin, grade 4 thrombocytopenia with carboplatin), these resolved within a week as expected after single agent chemotherapy. Moreover, they were within the anticipated toxicity profile of these chemotherapeutics when given as single agents. For example, in a study of 470 dogs with appendicular OSA receiving carboplatin and/or doxorubicin based chemotherapy, 4.1 and 9.6% of treated dogs experienced grade 3 and 4 AEs, respectively [30]. Importantly, in the current study only low grade hematologic and gastrointestinal AE’s predominated and the incidence of AEs was not increased beyond that expected with doxorubicin or carboplatin alone. Together, these pilot data support the notion that oclacitinib can likely be safely administered with standard maximum-tolerated dose chemotherapy in tumor-bearing dogs, without alterations in dose intensity or compounding toxicity.

There are several inherent weaknesses associated with this study. The small number of patients enrolled, lack of a contemporaneous prospective control group and the high drop-out rate due to progressive disease may have resulted in the study not capturing the full spectrum of adverse events possible with this combination therapy. Additionally, the short duration of this pilot study did not address the potential for any long-term adverse events associated with oclacitinib/chemotherapy combination therapy. One dog enrolled in this pilot study receive a lower dose of drug (0.25 mg/kg bw/dose). The authors feel that inclusion of this case is worthwhile because although the dose was low, the drug was still given in combination and even at the low dose provides drug exposure that inhibits relevant pathways. This assertion is based on both published data as well as the safety studies performed by Zoetis in beagle dogs in which lowest observed adverse effect level was oclacitinib 0.25 mg/kg bw/dose (0.5 mg/kg bw/day) based on hypocellularity of lymphoid and hematopoietic tissues. The hypocellularity of lymphoid/hematopoietic tissues is consistent with JAK inhibition. With respect to response assessment, the heterogeneity of tumor histologies enrolled precludes vigorous assessment of tumor types that may be more amenable to oclacitinib/chemotherapy combinations. Finally, as only two cytotoxic chemotherapies were assessed in this study, our findings may not apply to all potential chemotherapy/oclacitinib combinations.

Given the importance of JAK/STAT signaling in the context of normal immune system functions, there has been concern that inhibition of this pathway may impair immune surveillance and increase the risk for tumor development. Meta-analyses of patients with rheumatoid arthritis treated with tofacitinib have found no statistically significant increased risk of development of malignancies even with long term use [29, 31, 32]. However, a recent study of patients with myeloproliferative disorders treated with ruxolitinib found a 16-fold increased risk of development of aggressive B-cell lymphomas, particularly in 3 of 4 patients who had a pre-existing clonal immunoglobulin gene rearrangement in the bone marrow as early as 6 years prior to the development of lymphoma [33]. The mechanism of this increased risk remains unclear, although it may be related to the immune effects of JAK1J/AK2-specific inhibition by ruxolitinib, or reduction of myeloid cells allowing for proliferation of malignant B cells with a pre-existing clonal expansion [33]. Interestingly, the effects of JAK1/2 inhibition were mirrored in *Stat1*^*−/−*^mice: 16 of 24 mice developed a spontaneous myeloid hyperplasia with the concomitant presence of aberrant B cells indicating a possible role for loss of STAT1 activity in the process of B cell neoplasia following ruxolitinib treatment [33].

Veterinarians often discontinue oclacitinib in the face of a cancer diagnosis, given the product label designation that it may exacerbate neoplastic conditions. A long term study of oclacitinib in 247 dogs found that 16 dogs were diagnosed with a confirmed or suspected malignancy, 10 of which were euthanized for their malignancy [18]. The relationship between oclacitinib and development of these malignancies remains unclear, as the cancers noted in these patients were not unexpected given the age and breed of the affected dogs. Additionally, four of the sixteen patients in this study had been on oclacitinib for less than 60 days prior to the diagnosis of their neoplasia, making a causative relationship unlikely in these patients. Another study of 283 oclacitinib treated dogs over a 112 day treatment period reported two mast cell tumors and one heart base mass developed during the study period, none of which were attributed to treatment [17]. Two shorter-term studies did not report the development of any malignancies during a 28 day study period [19, 20]. Taken together, the published data regarding the incidence of neoplasia in dogs with atopy that receive oclacitinib does not clearly indicate a causative or correlative effect. It is also important to note that at least in human populations, patients with immune dysregulation, such as rheumatoid arthritis, are at increased risk for neoplasia compared to the population of unaffected patients. Therefore, when assessing the incidence of cancer within a patient population treated with tofacitinib for rheumatoid arthritis, it is necessary to compare data with a similar population of patients with rheumatoid arthritis that did not receive tofacitinib to develop accurate standardized incidence ratios. Such comparisons have not yet been undertaken in veterinary medicine.

No objective responses were noted in this study. Although JAK1/JAK2 inhibitors have been shown to inhibit growth of canine lymphoma cells and malignant mast cells in vitro, there is no published data regarding the effects of such JAK inhibitors on canine tumor cell lines derived from solid tumors [34, 35]. Most of the patients enrolled in this study were at advanced stages of disease with tumor types not widely regarded to be chemo-responsive. In contrast to the success of these JAK inhibitors in human myeloproliferative neoplasia, early-phase clinical trials of JAK inhibitors in solid tumors have been disappointing thus far [10, 36]. With respect to the role of STAT3 in solid tumors, constitutive activation is common but it remains unclear if this is a specific driver of tumor growth or if they are secondary to other changes such as upstream cytokine signaling [7, 11]. Moreover, it is possible that dysregulated JAK/STAT3 signaling has its greatest impact within the context of tumor promotion through modulation of the local microenvironment [27]. To date, there have been no human clinical trials of JAK/STAT inhibitors in combination with chemotherapy and as such, the potential for therapeutic synergy has not yet been explored investigated in human cancer therapy.

## Conclusions

This pilot study demonstrates that oclacitinib administered in combination with MTD doxorubicin or carboplatin chemotherapy is safe and well-tolerated in dogs with advanced solid tumors. While no objective responses were noted, no patients were withdrawn due to adverse events or toxicity. These data lay the groundwork for future studies exploring the biologic activity of combined oclacitinib/cytotoxic chemotherapy in dogs with cancer that may be less advanced/drug resistant and therefore more amenable to therapeutic intervention.

## Data Availability

The datasets used and/or analyzed during the current study are available from the corresponding author upon reasonable request.

## Abbreviations

AE: Adverse event
ALP: Alkaline phosphatase
ALT: Alanine transferase
ASACA: Anal sac adenocarcinoma
AST: Aspartate aminotransferase
CR: Complete response
ECOG: Eastern cooperative oncology group
EPO: Erythropoeitin
FDA: Food and Drug Administration
FS: Female spayed
GM-CSF: Granulocyte-monocyte colony stimulating factor
JAK: Janus kinase
MC: Male castrated
MTD: Maximum tolerated dose
OMM: Oral malignant melanoma
OSA: Osteosarcoma
PD: Progressive disease
PO: Per os
PR: Partial response
RECIST: Response evaluation criteria in solid tumors
SD: Stable disease
STAT: Signal transducer and activator of transduction
TPO: Thrombopoeitin
VCOG-CTCAE: Veterinary cooperative oncology group common terminology criteria for adverse events
